# Side-firing intraoperative ultrasound applied to resection of pituitary macroadenomas and giant adenomas: A single-center retrospective case-control study

**DOI:** 10.3389/fonc.2022.1043697

**Published:** 2022-11-30

**Authors:** Katherine E. Baker, Austin C. Robbins, Robert G. Wasson, Martin G. McCandless, Seth T. Lirette, Rebekah J. Kimball, Chad W. Washington, Gustavo D. Luzardo, Scott P. Stringer, Marcus A. Zachariah

**Affiliations:** ^1^ Department of Neurosurgery, The University of Mississippi Medical Center, Jackson, MS, United States; ^2^ Department of Data Science, The University of Mississippi Medical Center, Jackson, MS, United States; ^3^ Department of Otolaryngology, The University of Mississippi Medical Center, Jackson, MS, United States

**Keywords:** adenoma, skull base, ultrasound, endoscopic, imaging, sella, tumor, neurosurgery

## Abstract

**Introduction:**

Multiple intraoperative navigation and imaging modalities are currently available as an adjunct to endoscopic transsphenoidal resection of pituitary adenomas, including intraoperative CT and MRI, fluorescence guidance, and neuronavigation. However, these imaging techniques have several limitations, including intraoperative tissue shift, lack of availability in some centers, and the increased cost and time associated with their use. The side-firing intraoperative ultrasound (IOUS) probe is a relatively new technology in endoscopic endonasal surgery that may help overcome these obstacles.

**Methods:**

A retrospective analysis was performed on patients admitted for resection of pituitary adenomas by a single surgeon at the University of Mississippi Medical Center. The control (non-ultrasound) group consisted of twelve (n=12) patients who received surgery without IOUS guidance, and the IOUS group was composed of fifteen (n=15) patients who underwent IOUS-guided surgery. Outcome measures used to assess the side-firing IOUS were the extent of tumor resection, postoperative complications, length of hospital stay (LOS) in days, operative time, and self-reported surgeon confidence in estimating the extent of resection intraoperatively.

**Results:**

Preoperative data analysis showed no significant differences in patient demographics or presenting symptoms between the two groups. Postoperative data revealed no significant difference in the rate of gross total resection between the groups (p = 0.716). Compared to the non-US group, surgeon confidence was significantly higher (p *< 0.001)*, and operative time was significantly lower for the US group in univariate analysis (p = 0.011). Multivariate analysis accounting for tumor size, surgeon confidence, and operative time confirmed these findings. Interestingly, we noted a trend for a lower incidence of postoperative diabetes insipidus in the US group, although this did not quite reach our threshold for statistical significance.

**Conclusion:**

Incorporating IOUS as an aid for endonasal resection of pituitary adenomas provides real-time image guidance that increases surgeon confidence in intraoperative assessment of the extent of resection and decreases operative time without posing additional risk to the patient. Additionally, we identified a trend for reduced diabetes insipidus with IOUS.

## Introduction

Pituitary adenomas comprise a group of tumors differing in cell origin, response to treatment, and function. Common symptoms include hormonal dysfunction, vision changes, and headaches (1). Maximal resection is associated with prolonged progression-free survival, improvement of neurological deficits, and an increased likelihood of hormonal remission (2–4). Several technologies are currently employed to aid in the resection of pituitary adenomas. Intraoperative neuronavigation may confirm visual identification of anatomy; however, its effectiveness may be limited by intraoperative tissue shift, especially during the resection of larger tumors. Intraoperative MRI (iMRI), intraoperative CT (iCT), and fluorescence guidance may also be used to maximize safe resection (5). However, these techniques are not always available and may substantially increase the time, cost, and complexity of pituitary surgery.

Intraoperative ultrasound (IOUS) has not commonly been used in pituitary adenoma resection but has recently become more prevalent (6–10). Both end-firing and side-firing probes are available, each suited for specific applications. End-firing probes are helpful for depth assessment, while side-firing probes enhance awareness of anatomy adjacent to the probe tip and potentially beyond the endoscopic field of view. Early generation end-firing ultrasound probes were larger, which limited the effectiveness of these models. In some cases, the size of these probes prevented the advancement of the probe tip into the sella turcica, restricting use to the sphenoid sinus (11). Recent models of both end-firing and side-firing probes have been designed specifically for use in transsphenoidal surgery. We have previously reported the potential benefits of end-firing IOUS technology in the resection of a clival chordoma (12).

**Figure 1:**
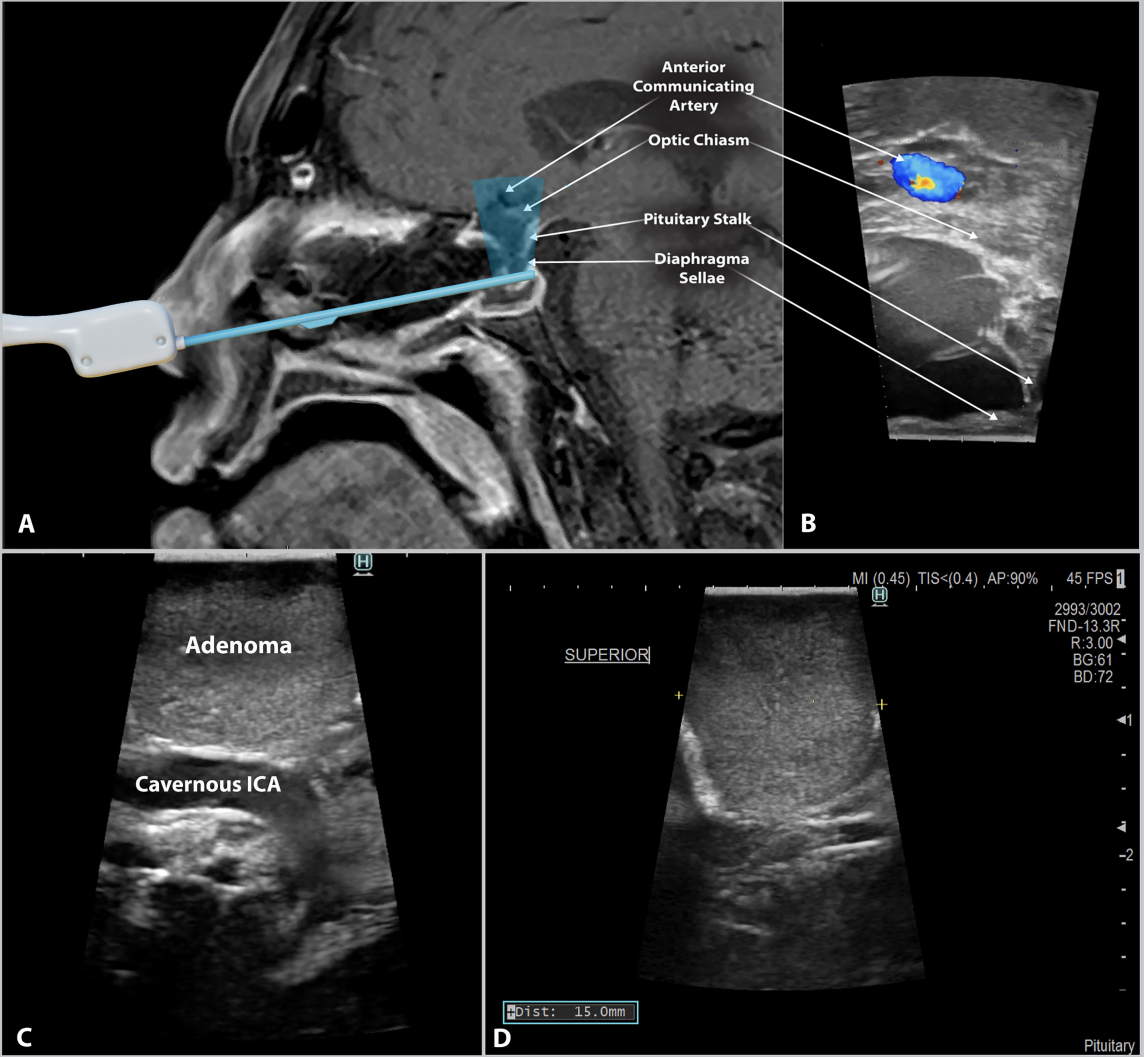
Side-firing intraoperative ultrasound in endoscopic endonasal pituitary surgery. **(A)** Schematic image depicting the scanning window of the side-firing ultrasound transducer. A digital ultrasound probe model is superimposed onto a T1 post-gadolinium MRI. **(B)** Intraoperative ultrasound image from the same patient showing intraoperative imaging of the surrounding parasellar anatomy. During image acquisition, the probe tip was abutted to the inferior surface of the diaphragma sellae, as demonstrated in Figure 1A. **(C)** Side-firing IOUS image showing pituitary adenoma tissue and the location of the cavernous segment of the Internal Carotid artery (cavernous ICA). The IOUS probe is directed laterally within the sella turcica. This image demonstrates the ability to identify critical structures and their relationship to the tumor tissue. **(D)** IOUS can be used to obtain tumor size data intraoperatively. The yellow symbols (+) in the above image indicate the location of the measurement, with the results displayed in the bottom left corner.

The development of relatively low-cost, minimally invasive, side-firing probes has allowed surgeons to use IOUS within the sella turcica for optimal imaging of the cavernous carotids and parasellar region. Side-firing IOUS may improve the surgeon’s ability to estimate the extent of resection while avoiding injury to nearby anatomy and perhaps improving the safety of endoscopic transsphenoidal resection of pituitary adenomas. These newer probes have proved helpful for identifying vascular structures, such as the internal carotid artery and branches of the Circle of Willis, in addition to other vital structures, such as the optic chiasm and diaphragm sellae (13) (**Figure 1**). The surgeon may also use other features of the IOUS to guide their resection. For example, measurements are easily obtained intraoperatively and can provide perspective on the size of the residual tumor and the distance to nearby structures (**Figure 1D**). Clear identification of these structures allows the surgeon to accurately assess their location and tailor the resection accordingly, thus preventing CSF leaks caused by violation of the diaphragm or damage to other nearby structures. Previous studies in the literature report the implementation of new imaging techniques in surgical settings and their effects on self-reported surgeon confidence when identifying key structures (14–20). However, the effects of IOUS guidance on surgeon confidence are not well described. Our study compares surgeon confidence with and without the use of side-firing IOUS and shows that side-firing IOUS guidance increases surgeon confidence.

## Methods

### Study design

A retrospective analysis was conducted on all patients admitted for elective endonasal transsphenoidal resection of pituitary adenomas by a single surgeon at the University of Mississippi Medical Center (UMMC) from 10/7/2020 to 2/23/2022. The study focused on the following data: patient demographics (age, sex, race), preoperative findings (presenting symptoms, tumor size, and Knosp grade), intraoperative findings (surgeon confidence, operative time, and complications), and postoperative findings (gross total resection, subtotal resection, complications, and length of stay). Patients underwent preoperative MRI with and without gadolinium contrast and preoperative hormone evaluations. Surgery for the non-US control group consisted of twelve (n = 12) patients who received surgery before implementing IOUS for pituitary macroadenoma resection at our institution on 7/13/2021. Following this date, all subsequent surgeries (n = 15) were guided by the Fujifilm/Hitachi side-firing pituitary guidance ultrasound transducer and neuronavigation.

### Surgical approach

An endoscopic endonasal transsphenoidal approach was performed on all patients in this study. The initial portion of the procedure and follow-up appointments were conducted in collaboration with Otolaryngology. The procedure was handed off to neurosurgery after entry into the sphenoid sinus, and the remainder of the surgery was performed following standard endoscopic techniques.

### Intraoperative ultrasound

The IOUS probe used in this study is the Fujifilm/Hitachi pituitary guidance transducer. The Fujifilm ultrasound probe is a commercially available, side-firing linear array transducer with a 60˚ trapezoidal scanning window and a maximum diameter of 2.87 mm. The probe fires at a 90-degree angle from the axis of insertion. The scanning window is tilted as the surgeon rotates the probe, and images are acquired perpendicular to the probe axis. This capability allows the surgeon to sweep through the surrounding anatomy and creates a large field of view that is particularly useful when working in the surgical corridor of endoscopic endonasal surgery (**Figure 1**). For the US group, the surgeon used IOUS several times as the case progressed to estimate the extent of resection and identify residual tumor. Additionally, color flow Doppler imaging was used to quickly assess proximity to intracranial vasculature (**Figure 1**).

### Outcome measures

Outcome measures used to assess the effectiveness of side-firing IOUS were the extent of tumor resection, postoperative complications, length of hospital stay (LOS) in days, operative time, and self-reported surgeon confidence in assessing the extent of resection intraoperatively. To measure surgeon confidence, the surgeon was asked at the end of each case to rate his confidence in the intraoperative assessment of the extent of resection. This measure is subjective and scored on a scale of 1-10, with 10 being the highest confidence and 1 being the lowest confidence. The extent of resection was determined based on the interpretation of each patient’s three-month postoperative MRI. GTR was defined by the absence of visible tumor tissue on three-month postoperative MRI as determined by a neuroradiologist blinded to the study.

### Data collection and analysis

UMMC’s institutional review board approved this study, and informed consent was obtained from all patients (IRB File # 2021-1012). Patient data were collected from the electronic medical record and managed using REDCap (Research Electronic Data Capture) electronic data capture tools hosted at the University of Mississippi Medical Center (19, 20). Data manipulation and visualization were performed using GraphPad Prism.

### Statistical analysis

Patient characteristics were analyzed with standard summary statistics. An alpha of 0.05 was selected as the threshold of significance for all analyses, and significant p values are denoted with an asterisk (*) in the figures. A X^2^-test or independent t-test was used to assess significance where appropriate, and Fisher’s Exact Test was used to assess significance in smaller subpopulations. Potential correlations were examined using linear regression and multilinear analysis to assess the multivariate interactions of surgeon confidence, tumor size, and operative time. Data is presented in this paper as mean ± standard deviation or percent of patients when appropriate.

## Results

### Demographic data

The case-control study included two groups of patients with pituitary adenomas. The first group underwent tumor resection without IOUS guidance (n = 12), and the second with IOUS guidance (n = 15). The non-US group consisted of 67% males and 33% females, 60% African American and 40% Caucasian, with an average age of (47.4 ± 16.9) years. The US group included 69% men, 31% women, 69% African American, and 31% Caucasian, with an average age of (57.3 ± 7.4) years. There were no significant differences in patient demographics (age, sex, race) between the two groups (**Table 1**).

**Table 1:** Baseline Characteristics and Presenting Symptoms.

Characteristics	US, n=15	Non-US, n=13	P-Value
Age at surgery (years)			0.067
Mean ± SD	57.3 ± 7.4	47.4 ± 16.9	
Range	42-66	19-74	
95% CI	53.3-61.4	37.2-57.6	
M/F (% Female) *	10/5 (33%)	9/4 (31%)	>0.99
AA/C (% AA) *	9/6 (40%)	9/4 (31%)	0.705

AA, African American; C, Caucasian; LOS, Length of Stay.

X^2^-test and independent t-test were performed.

*Fisher Exact Test was performed.

Bold text indicates P < 0.05.

### Preoperative tumor characterization

**Figure 2:**
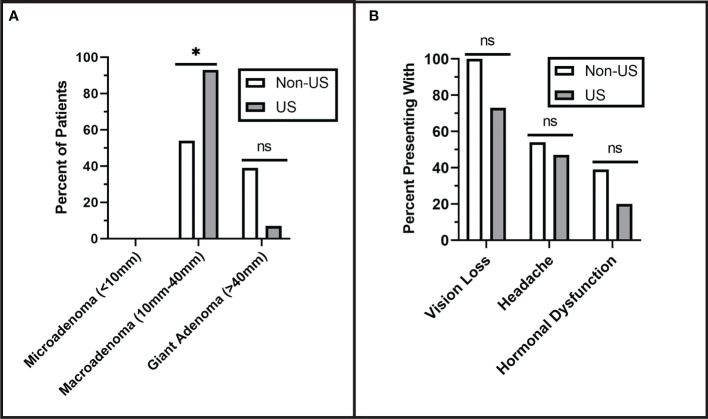
Preoperative Tumor Characterization. **(A)** Pituitary adenomas were classified by size. No microadenomas were observed in either group. The US group had significantly more macroadenomas than the non-US group (p = 0.029). **(B)** There were no significant differences in the rates of presenting symptoms including vision loss (p = 0.102), headache (p = 0.705), and hormonal dysfunction (p = 0.410). Asterisks (*) indicate significance of p < 0.05. Values that did not reach the threshold for significance (p= <0.05) were labeled as non-significant (ns).

Tumors were characterized preoperatively for both groups and classified as microadenoma (<1 cm), macroadenoma (1 cm- 4 cm), or giant adenoma (> 4 cm). None of the patients had microadenomas, 78% had macroadenomas, and the remainder were giant adenomas (22%). There was no significant difference in tumor size between the non-US group (3.43 ± 1.5 cm) and the US group (2.89 ± 1.5 cm), although the US group had a greater proportion of macroadenomas (93%) than the non-US group (54%) (p = 0.029). The difference in tumor size between groups was controlled for in subsequent analyses of operative time, surgeon confidence, and tumor size presented below. There was no significant difference in preoperative Knosp Grade, as shown in **Figure 2**. Patients in both groups had similar presenting symptoms, with the most common being vision loss (non-US: 100%, US: 73%), followed by headache (non-US: 54%, US: 47%), and hormonal dysfunction (non-US: 39%, US: 20%) (**Figure 2**).

### Postoperative results

**Figure 3:**
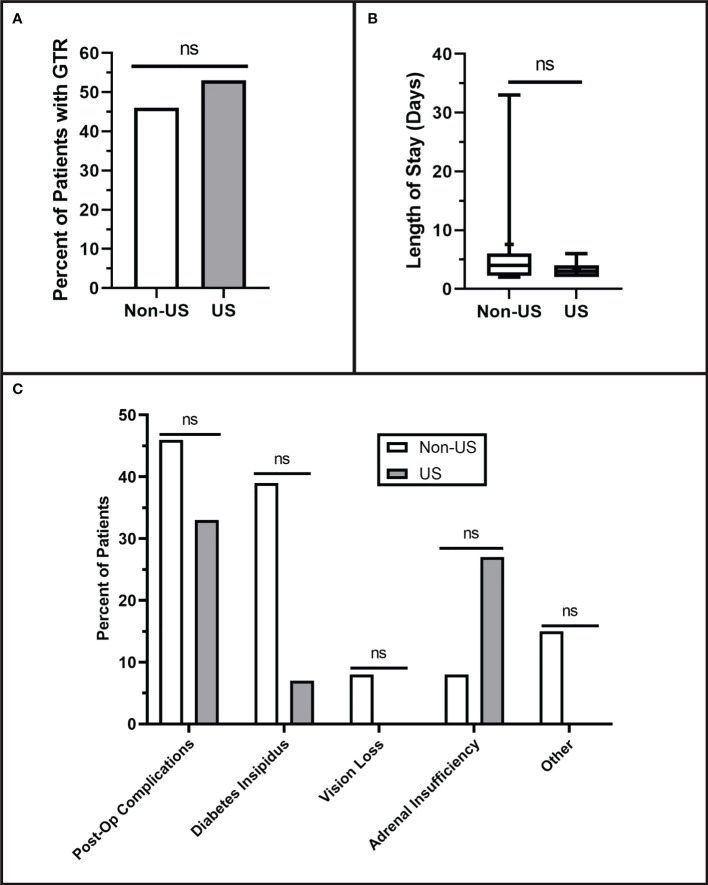
Postoperative Resection Results. **(A)** Qualitative extent of tumor resection was classified as either gross total (GTR) or subtotal. The extent of resection was determined based on the interpretation of each patient’s three-month postoperative MRI. No differences were observed in qualitative extent of resection (p = 0.716). **(B)** Length of hospital stay showed no difference between the groups (p = 0.155). **(C)** There were no significant differences in the numbers of postoperative complications (p = 0.488) including diabetes insipidus (p = 0.069), vision loss (p = 0.464), adrenal insufficiency (p = 0.333), or other complications (P = 0.206). Values that did not reach the threshold for significance (p= <0.05) were labeled as non-significant (ns).

IOUS use did not affect the extent of resection; gross total resection was achieved in 53% of US patients and 46% of non-US patients (p = 0.716). Within the subset of patients with subtotal resection, the postoperative Knosp grades showed no difference, as shown in **Figure 3** (p = 0.343). There was no difference in total postoperative complications between the two groups (non-US: 46%, US: 33%) (p = 0.488). However, there was a trend toward fewer diabetes insipidus complications in the US group (7%) compared to the non-US group (39%) (p = 0.069), although this did not reach our threshold for statistical significance. More data will need to be collected to confirm this trend. There was no difference in postoperative length of hospital stay between the non-US group (7.08 ± 9.3 days) and US group (3.13 ± 1.3 days) (p = 0.155).

**Figure 4:**
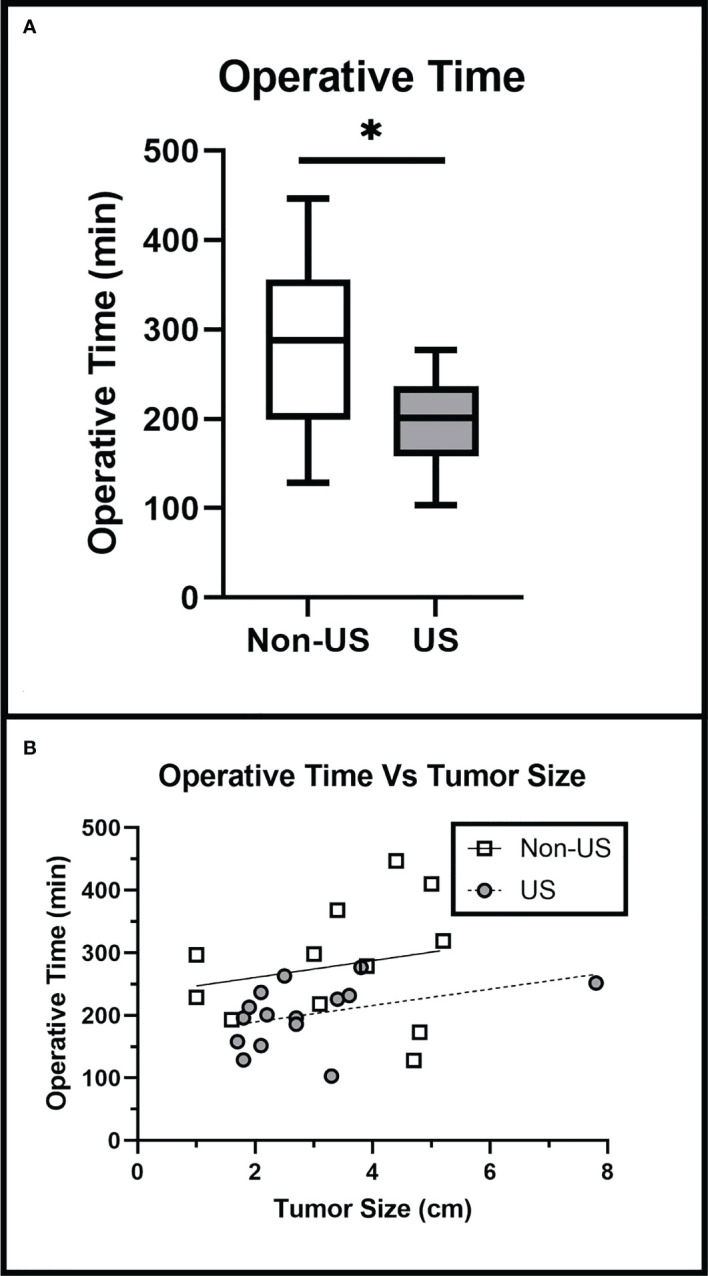
Operative Time is Reduced with the Use of IOUS: **(A)** IOUS significantly reduced procedure duration (p = 0.011). **(B)** IOUS reduced operative time when adjusted for tumor size (Slope p = 0.7991, Intercept p < 0.0001). Asterisks (*) indicate a significance of p < 0.05. Crosses (+) indicate mean values.

Operative time was significantly lower in the US group (201 ± 48 minutes) than in the non-US group (280 ± 93 minutes) (p = 0.011). Linear regression showed that the operative time remained lower in the US group than the non-US group when adjusted for tumor size (Slope p = 0.9844, Intercept p = 0.02) (**Figure 4**), suggesting that IOUS results in shorter operative time for all tumor sizes.

**Figure 5:**
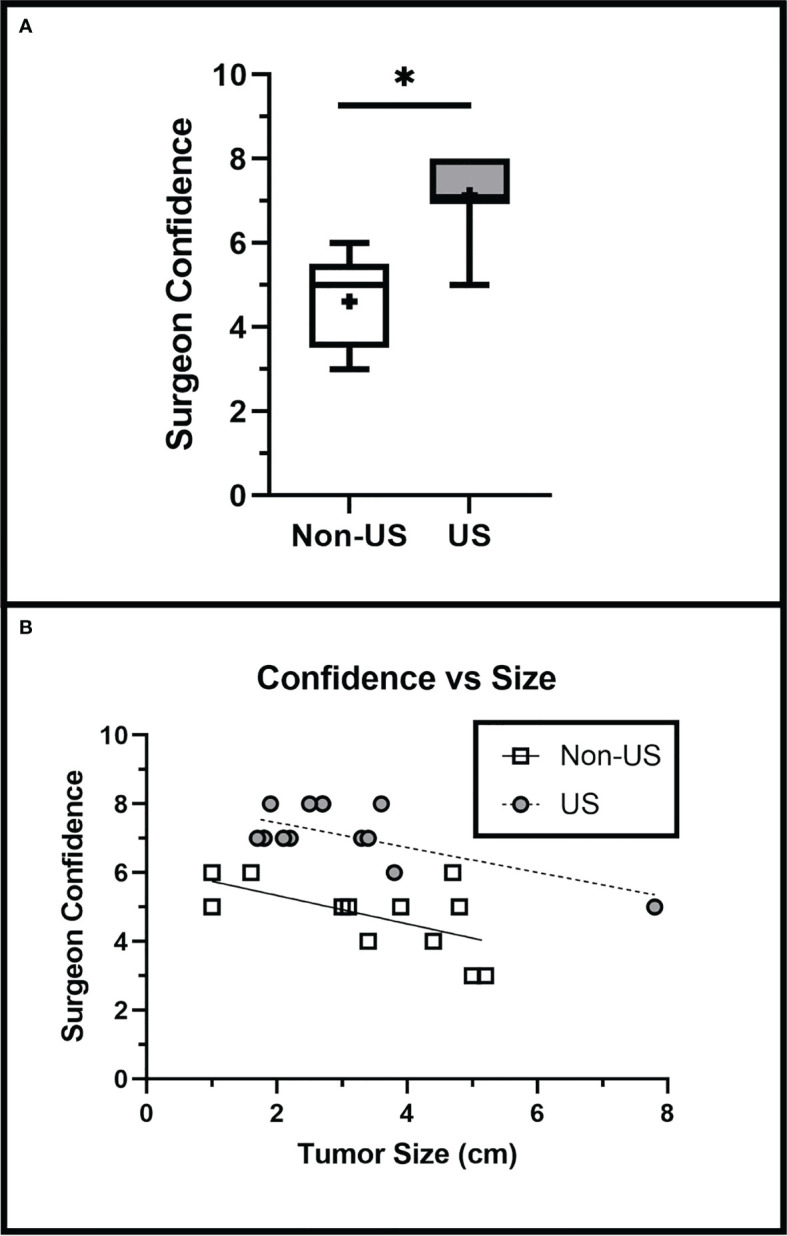
Surgeon Confidence is Increased with IOUS Use. **(A)** IOUS improved surgeon confidence in assessing the extent of tumor resection intraoperatively (p < 0.001). **(B)** Surgeon confidence was greater in the US group when adjusted for tumor size (Slope p = 0.7991, Intercept p = 0.02). Asterisks (*) indicate significance of p < 0.05. Crosses (+) indicate mean values.

In our study, surgeon confidence is a self-reported measure that we defined as how confident the surgeon feels in the accurate intraoperative assessment of extent of resection. The US group had a significantly higher average surgeon confidence level (6.9 ± 1.4) than the non-US group (4.9 ± 1.2) (p < 0.001). Surgeon confidence remained significantly greater in the US group than in the non-US group when adjusted for tumor size (Slope p = 0.7991, Intercept p < 0.0001) (**Figure 5**), indicating that IOUS use increased surgeon confidence regardless of tumor size.

**Figure 6:**
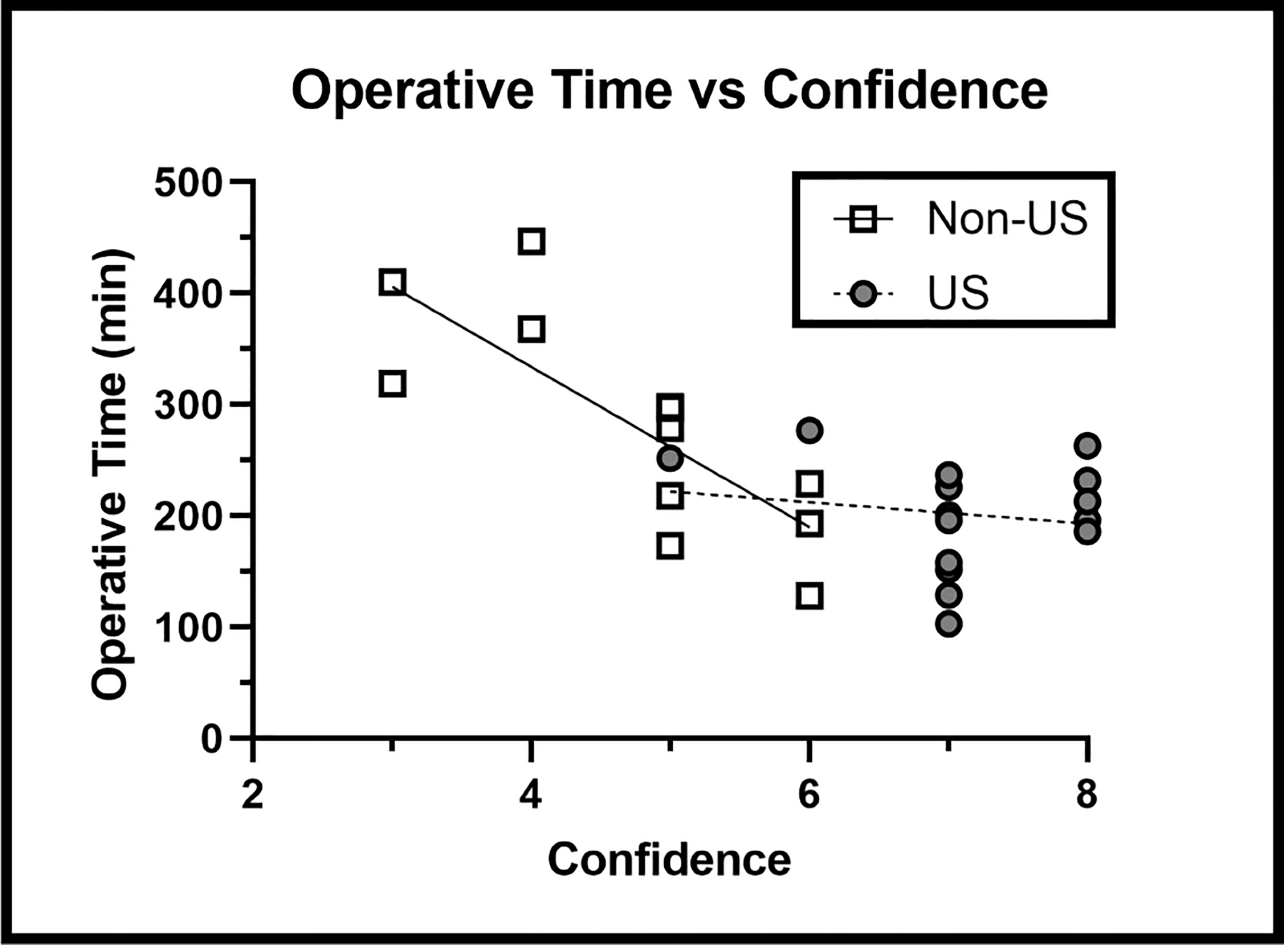
Operative Time versus Surgeon Confidence Findings: IOUS use prevented the increase in operative time associated with lower levels of surgeon confidence (p = 0.0168).

Without IOUS, operative time dramatically increased as surgeon confidence declined (R = -0.867); however, IOUS use did not show a significant increase in operative time associated with lower confidence (R = -0.223). IOUS use significantly reduced the increase in operative time associated with lower surgeon confidence in the non-US group (Slope p = 0.0168) (**Figure 6**), suggesting that IOUS speeds up operative times even when surgeon confidence levels are lower.

## Discussion

Intraoperative imaging technologies are implemented to provide guidance for a safer and more complete resection. Conventional intraoperative imaging techniques, such as neuronavigation and iMRI, may help optimize the resection of pituitary adenomas, although these modalities often have limitations (5, 7, 14, 18, 21, 22).

Neuronavigation is frequently utilized for preoperative planning and evaluation of the patient’s anatomy. However, as resection proceeds, intraoperative tissue shift may alter the anatomy of the surgical field and render preoperatively identified landmarks inaccurate. In the case of large and giant pituitary adenomas, the diaphragma sellae is often displaced from its usual location as the tumor expands superiorly. As resection proceeds, the diaphragma sellae descends from its preoperative location and can no longer be accurately localized on intraoperative imaging. Undetected tissue shift increases the risk of incomplete resection and the risk of injury to critical structures. To address this concern, iMRI has become increasingly widespread; however, iMRI is costly, time-intensive, may require modification of the operating room layout to accommodate the equipment, and has been associated with increased rates of false-positive identification of tumor tissue (5, 21). iMRI, in particular, substantially prolongs overall procedural time because of the time associated with operation of the iMRI machine and image acquisition (23–26). Additionally, fluid accumulation in and around the parasellar region may complicate the interpretation of MR images during resection (7).

iCT is well-described in both adult and pediatric neurosurgery. iCT is associated with increased operative times, although it is considerably faster than iMRI (26). iCT provides high-quality images that can be beneficial in specific pathologies but does not provide the soft tissue imaging resolution afforded by iMRI. Further, iCT may increase radiation exposure to patients and operating room staff (27, 28).

Fluorescent label-based guidance may improve resection by selectively causing tumor tissue to fluoresce, assisting visualization and resection. Studies have shown conflicting results among the available fluorescent agents (19, 29). Sodium fluorescein (FNa) and 5-ALA with laser-based optical biopsy are two agents that have been shown to selectively fluoresce adenomatous tissue; however, these results are not consistent across all studies (29). In a study specific to endoscopic endonasal skull base surgery, 5-ALA was ineffective for the identification of neoplastic pituitary adenoma tissue (19). Another selective fluorescent agent, OTL38, exhibits promise in non-functioning pituitary adenomas. Newer agents, such as OTL38, are near-infrared region (NIR) fluorophores, while older agents, such as 5-ALA, are visible-light fluorophores. OTL38 binds to folate-expressing cells of non-functional adenomas and has greater photon tissue penetration than visible light fluorophores, allowing clearer demarcation between normal and neoplastic tissue (30). Although, this agent is still under investigation and more evidence is necessary to distinguish these agents as effective selective fluorescent agents to guide the resection of pituitary adenomas (29, 30).

IOUS has previously been used as an adjunct technology in endonasal pituitary surgery; however, the large size of older probes and limited availability have prevented widespread use in pituitary surgery. Recent probe advancements, particularly those designed specifically to suit the endoscopic endonasal approach, have allowed IOUS to become a much more effective tool in the transsphenoidal resection of pituitary adenomas. IOUS provides high-resolution real-time feedback to the surgeon without exposing the patient to additional radiation.

The side-firing IOUS enables the surgeon to quickly identify structures such as the diaphragma sellae, suprachiasmatic cistern, and cavernous carotids (**Figure 1C**). The surgeon may also utilize the intraoperative measuring capability of the probe to provide perspective on the size of the residual tumor and the distance to nearby structures (**Figure 1D**). Detection of critical structures with IOUS allows the surgeon to assess their location and limit their resection accordingly to prevent disruption of the nearby anatomy. For example, CSF leaks may be provoked by violating the diaphragma sellae during the transsphenoidal resection of pituitary adenomas.

Previous studies of IOUS have reported decreased incidence of intraoperative complications and intraoperative bleeding (8, 9). Interestingly, our results demonstrated a trend toward decreased postoperative diabetes insipidus with IOUS, which was not noted in previous studies of side-firing IOUS. These results are likely due to the increased confidence in identifying normal pituitary tissue with the IOUS probe and the ability to avoid disruption of the posterior pituitary gland and/or pituitary stalk, similar to the previous example of the diaphragma sellae. Additionally, pituitary adenomas may contain intratumoral membranes or cystic components, which may be mistaken for the diaphragma sellae. IOUS may be used to prevent this misidentification and ensure appropriate resection of tumor tissue concealed behind the membrane or cyst wall.

This study attempts to perform an initial quantification of the benefits of side-firing IOUS in pituitary surgery in a controlled manner. According to our data, IOUS has no negative impact on patient outcomes and is associated with similar resection fraction, complications, and length of stay compared to control. Additionally, IOUS shortens the operative time and increases surgeon confidence. These results indicate that IOUS is a safe, effective, and efficient adjunct to endoscopic endonasal resection of pituitary adenomas.

However, there are limitations to IOUS use in endoscopic endonasal surgery. Some neurosurgeons have little experience using ultrasound in the operating room, so they must undergo IOUS training which takes time and practice to develop confidence when interpreting US images intraoperatively (6). The US machine occupies space in the operating room and may require repositioning other equipment and alteration of the workflow. However, the IOUS machine requires far less space than iCT or iMRI machines. Another drawback to IOUS use is the cost of the specialized probe, IOUS machine, and necessary training (16). While this study demonstrates the benefits of a side-firing US probe for transsphenoidal resection of large macroadenomas in the parasellar region, end-firing probes may be more appropriate in some circumstances. In the case of tumors that displace the normal pituitary posteriorly, an end-firing probe would be better indicated to properly visualize the posteriorly displaced pituitary gland to avoid its injury. If the pituitary is translated superiorly, a side-firing probe is more beneficial. This case typically reveals a diaphragm with a thickened appearance on IOUS due to the superior displacement of the normal pituitary gland, which adheres the gland to the diaphragm. Other surgeons have reported success using the end-firing probe to find small microadenomas within normal pituitary (4, 11, 31). One limitation of the study is the higher percentage of giant adenomas in the non-US group, although there was no significant difference in GTR between the groups. As tumor size and pattern of extension are key factors in achieving GTR, future studies between giant adenomas with similar patterns of extension and tumor characteristics are needed to resolve this limitation.

Despite these limitations, this study demonstrated that IOUS is associated with reduced operative time and increased surgeon confidence in assessing the extent of resection intraoperatively. Because surgeon confidence is subjective, the results may differ between surgeons. Additional studies are needed to explore how side-firing IOUS guidance impacts surgeon confidence among a larger group of surgeons. IOUS may enhance understanding of the intraoperative normal and tumor anatomy, allowing the surgeon to feel more confident as they make surgical decisions. The surgeon can employ the IOUS probe before proceeding with resection to confirm surgical orientation and location of critical structures. Before completion of the procedure, the probe may be used to verify that all tumor has been resected.

## Conclusion

Existing adjunct technologies face limitations in resection of large and giant pituitary adenomas. This case-control study demonstrated that IOUS decreased operative time and increased surgeon confidence without any negative impact on patient outcomes. Additionally, our data suggested a nonsignificant trend towards decreased incidence of postoperative diabetes insipidus, which may potentially result from increased confidence in identifying normal pituitary tissue and avoiding injury to the posterior pituitary. In further studies, a change in surgical outcomes may be observed with larger sample sizes. Our findings suggest that IOUS is a valuable adjunct to guide resection of large and giant pituitary adenomas.

## Data availability statement

The raw data supporting the conclusions of this article will be made available by the authors, without undue reservation.

## Ethics statement

The studies involving human participants were reviewed and approved by University of Mississippi Institutional Review Board. The patients/participants provided their written informed consent to participate in this study.

## Author contributions

KB drafted the manuscript, prepared figures and figure legends, operated ultrasound machine during resection, collected intraoperative ultrasound images and conducted chart review/data collection. AR provided writing assistance, assisted/operated ultrasound machine during resection, collected intraoperative ultrasound images, and conducted chart review/data collection. RW drafted the manuscript, conducted statistical analyses, prepared figures and figure legends. MM conducted statistical analyses and provided direction, SL provided guidance for statistical analysis, RK provided writing assistance and direction. SS collaborated with MZ to perform the endoscopic endonasal surgery. MZ provided care to the patient, collected intraoperative ultrasound images, and provided important critical feedback on intellectual content. All authors read and approved the manuscript. All authors contributed to the article and approved the submitted version.

## Funding

This work was supported by research grants from Fujifilm and from the Mississippi Center for Translational and Clinical Research (U54GM115428).

## Conflict of interest

Author MZ owns stock in Exelixis and Zinnia Healthcare, for whom he has also consulted. He has participated in prior research projects that were supported by BK Medical. He also has research support from the University of Mississippi Medical Center for clinical and translational research.

The remaining authors declare that the research was conducted in the absence of any commercial or financial relationships that could be construed as a potential conflict of interest.

## Publisher’s note

All claims expressed in this article are solely those of the authors and do not necessarily represent those of their affiliated organizations, or those of the publisher, the editors and the reviewers. Any product that may be evaluated in this article, or claim that may be made by its manufacturer, is not guaranteed or endorsed by the publisher.
